# Nano-Biotechnology for Bacteria Identification and Potent Anti-bacterial Properties: A Review of Current State of the Art

**DOI:** 10.3390/nano13182529

**Published:** 2023-09-10

**Authors:** Shimayali Kaushal, Nitesh Priyadarshi, Priyanka Garg, Nitin Kumar Singhal, Dong-Kwon Lim

**Affiliations:** 1 KU-KIST Graduate School of Converging Science and Technology, Korea University, 145 Anam-ro, Seongbuk-gu, Seoul 02841, Republic of Korea; its.smiley.kaushal@korea.ac.kr; 2 National Agri-Food Biotechnology Institute (NABI), Sector-81, Mohali 140306, India; nitesh@nabi.res.in (N.P.); garg.priyanka@nabi.res.in (P.G.); 3 Department of Integrative Energy Engineering, College of Engineering, Korea University, 145 Anam-ro, Seongbuk-gu, Seoul 02841, Republic of Korea; 4 Brain Science Institute, Korea Institute of Science and Technology (KIST), 5, Hwarang-ro 14-gil, Seongbuk-gu, Seoul 02792, Republic of Korea

**Keywords:** bacteria identification, nanotechnology, sepsis, anti-bacterial activity, antimicrobial resistance

## Abstract

Sepsis is a critical disease caused by the abrupt increase of bacteria in human blood, which subsequently causes a cytokine storm. Early identification of bacteria is critical to treating a patient with proper antibiotics to avoid sepsis. However, conventional culture-based identification takes a long time. Polymerase chain reaction (PCR) is not so successful because of the complexity and similarity in the genome sequence of some bacterial species, making it difficult to design primers and thus less suitable for rapid bacterial identification. To address these issues, several new technologies have been developed. Recent advances in nanotechnology have shown great potential for fast and accurate bacterial identification. The most promising strategy in nanotechnology involves the use of nanoparticles, which has led to the advancement of highly specific and sensitive biosensors capable of detecting and identifying bacteria even at low concentrations in very little time. The primary drawback of conventional antibiotics is the potential for antimicrobial resistance, which can lead to the development of superbacteria, making them difficult to treat. The incorporation of diverse nanomaterials and designs of nanomaterials has been utilized to kill bacteria efficiently. Nanomaterials with distinct physicochemical properties, such as optical and magnetic properties, including plasmonic and magnetic nanoparticles, have been extensively studied for their potential to efficiently kill bacteria. In this review, we are emphasizing the recent advances in nano-biotechnologies for bacterial identification and anti-bacterial properties. The basic principles of new technologies, as well as their future challenges, have been discussed.

## 1. Introduction

Sepsis is a life-threatening health disorder that is hard to spot based on the symptoms. It is especially hard to spot it in neonates, young children, or people with health problems. Sepsis happens when the immune system overreacts to an infection and starts to damage tissues and cause organ dysfunction. [1,2,3,4]. As per the 2021 report, sepsis leads to mortality in intensive care units, with around 20% of deaths occurring annually and affecting about 48.9 million patients worldwide [5]. The most common cause of sepsis is a bacterial infection. Currently, the most preferred method for the treatment of bacterial infections is the use of antibiotics, as they have a quick outcome and are powerful as well as cost-effective. As the mortality rate is increasing worldwide due to pathogenic bacterial infections, more and more antibiotics are being misused in the healthcare system [6]. More importantly, the excessive use of antibiotics led to the emergence of super-bacteria, which could not be destroyed with conventional antibiotics [7,8,9]. Therefore, antibiotics should be carefully selected to minimize abuse.

**Figure 1:**
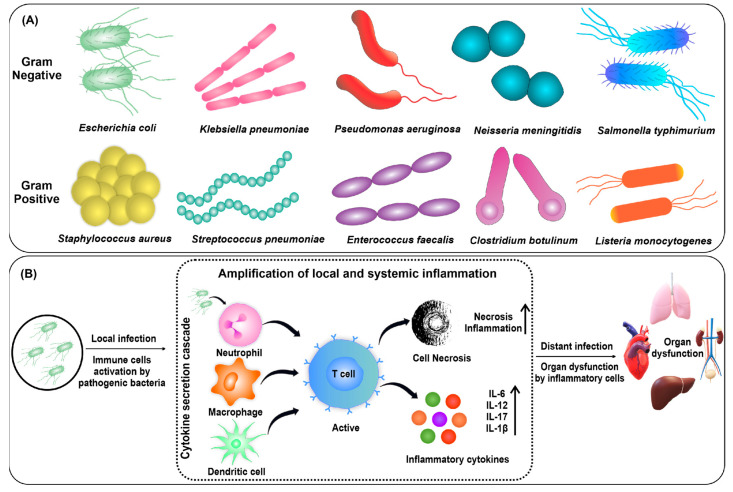
(**A**) The representative Gram-positive and Gram-negative bacteria causing sepsis; (**B**) the mechanism of sepsis. Reproduced with permission from [20]. Copyright 2018, Wiley.

The ten most common bacteria causing sepsis are *Escherichia coli* [10], *Staphylococcus aureus* [11], *Klebsiella pneumonia* [12], *Pseudomonas aeruginosa* [13], *Streptococcus pneumoniae* [14], *Enterococcus faecalis* [15], *Neisseria meningitidis* [16], *Salmonella typhimurium* [17], *Clostridium botulinum* [18], and *Listeria monocytogenes* [19]. In sepsis, both Gram-positive and negative bacteria play a key role in causing infection by secreting toxins (Figure 1A). These toxins generated by the bacteria stimulate the immune system in the host, which causes a cytokine storm and leads to organ dysfunction (Figure 1B) [20]. Since this is a very acute process, the bacteria should be cleared rapidly from the patient’s blood before sepsis occurs [21]. The primary way to minimize the occurrence of sepsis is the early identification of bacteria in the patient’s blood, which is then treated with proper antibiotics [22]. However, early identification is not easily attainable because of the time-consuming procedures of conventional culture-based methods [23,24]. The developed polymerase chain reaction (PCR)-based method by ROCHE (Septifast^®^) is also unsatisfactory because of its low accuracy for bacteria identification. Due to their genomic complexity, some bacterial species may be more difficult to detect by PCR-based methods. If these sequences are too complex or similar to those in other bacteria, the primers may not be specific enough to differentiate between different bacterial species [25]. Septifast^®^ can detect up to 25 pathogens related to sepsis [26]. A study on the ROCHE SeptiFast^®^ PCR system found low sensitivity (90.2%) and specificity (72.9%), which is not suitable for rapid and accurate bacterial identification [27].

Recently developed nanotechnology has shown great potential to solve the drawbacks of conventional methods for bacterial identification and treatment [28,29,30,31]. The distinct properties of nanomaterials have been exploited to efficiently identify and eradicate bacteria in everyday life and clinical settings. Based on the unique properties of surface plasmon resonance (SPR) [32], various optical identification methods, such as colorimetric [33], fluorometric [34], and spectrometric [35], have been developed. Along with a new strategy relying on the optical property of plasmonic nanomaterials, deep-learning-based data analysis further proved the strong capability to identify the types of bacteria [36].

In addition, it has been known that the anti-bacterial properties of various metallic nanoparticles such as gold, silver, zinc oxide, titanium, and copper exhibit anti-bacterial properties based on diverse mechanisms [37,38,39,40,41,42,43,44]. The unique property of metallic nanomaterials could further enhance the anti-bacterial property by incorporating new designs of materials in the nanoscale or incorporating antibiotics for targeted drug delivery [45,46], which can efficiently remove pathogenic bacteria and minimize the use of antibiotics. In this regard, the development of nanotechnology-based tools for bacteria identification and anti-bacterial action is a crucial area that requires further attention.

## 2. Methods to Identify Bacteria Species

A number of techniques are available for bacterial detection in clinical settings, but each one has its own scope and limitations. Some of the most common techniques used for bacterial detection and identification have been summarized in the following Table 1, along with their scope and limitations, which are discussed in detail in the subsequent section.

### 2.1. Culture Based Bacteria Identification

**Figure 2:**
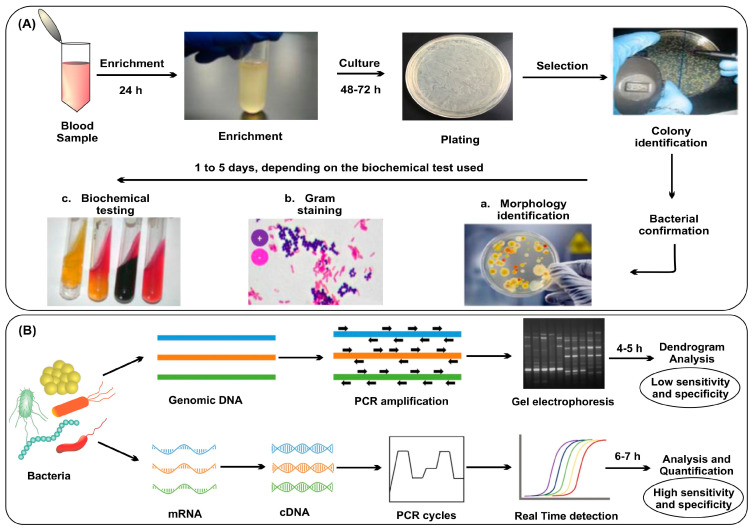
Methods for bacterial identification with (**A**) culture-based method, (**B**) PCR-based method. Reproduced with permission from [47,54]. Copyright 2020, Wiley; copyright 2020, Springer.

The culture-based method has been considered to be the most established and reliable method for bacterial identification. The culture-based method involves bacterial sample culturing in a laboratory setting using growth media and identification of bacteria on the basis of their morphology, Gram staining, and biochemical testing (Figure 2A) [47]. There are several advantages to culture-based methods, including the ability to detect a wide range of bacterial species, their adaptability, and their availability in most clinical laboratories. When used appropriately, this method can provide accurate and reliable identification results and also allow for antibiotic susceptibility testing [48]. Several factors are responsible for accurate identification results in culture-based methods, such as media type, quality of the sample, and handling skills. However, the main limitation of the culture-based method is its time-consuming nature, taking up to several days to obtain results, which may delay diagnosis and treatment, leading to poor patient outcomes [49]. Moreover, culture-based methods have limited sensitivity, and some bacterial species may be difficult to culture, leading to false-negative results. Most of the pathogenic bacteria can grow within the time period of 24 h, but some of them can take up to days for their visible growth on the culture plates [50]. Another factor is cost, which may increase when specialized media and equipment are needed for specific bacteria. The culture-based method is also susceptible to contamination, leading to false-positive results. A recent meta-analysis was conducted to compare the clinical outcomes of culture-positive and culture-negative patients, out of which only about 40.1% of the patients having sepsis or septic shock showed a positive blood culture. A similar mortality rate was observed in both culture-positive and culture-negative patients, which demonstrated the unreliability of blood cultures [51]. Antimicrobial therapy before the blood culture can also result in a negative blood culture, which decreases the bacterial identification probability [52]. The future challenges of culture-based methods include limitations in detecting fastidious or slow-growing bacteria, the increasing demand for rapid diagnostic techniques, and the emergence of antibiotic-resistant strains that may not grow on standard culture media.

### 2.2. PCR-Based Bacterial Identification

PCR is a technique used for the amplification of a unique DNA segment from a complex mixture of genetic material. PCR has been widely used in bacterial identification, especially in clinical and research settings. The principle of PCR in bacterial identification relies on bacterial DNA amplification by using specific primers that target conserved regions of the bacterial genome [53]. The PCR should be optimized to minimize non-specific amplification, which can otherwise lead to false positives and decreased sensitivity. PCR has several advantages, including high sensitivity, specificity, and speed. The bacterial DNA can be quantified by real-time PCR during the amplification process, where the amplification of the targeted amplicon is directly proportional to the fluorescent emission of a dye that generally binds to the amplicon. This allows the detection of bacterial infections at a very early stage. The amplified product of target DNA can be identified using gel electrophoresis at the end or in real time by a fluorescence signal indicating the presence or absence of DNA fragments of the target (Figure 2B) [54]. Multiplex PCR offers simultaneous detection of multiple organisms in one go by using different primers for the amplification, which increases the speed and efficiency of bacterial identification [55]. Despite several advantages, the PCR technique also presents some limitations in this field. Potential for contamination is one of the major limitations of this technique, which can occur at any stage of sample preparation, the amplification process, or the analysis step, leading to decreased sensitivity or false positives. Another limitation of PCR is the possibility of false positives due to the presence of non-specific amplification products. The complexity of bacterial genome sequences can be a challenge for PCR identification, particularly in cases where the target sequence is highly conserved and present in multiple bacterial species [56]. In such cases, the primers designed for PCR amplification may not be specific enough to differentiate between closely related bacterial species or strains. Another challenge in PCR identification of bacteria is the presence of multiple copies of the target sequence in the bacterial genome, which can result in false positives or overestimations of bacterial load [57]. Many modifications to the PCR have been conducted to date to improve its identification performance. Septifast is an approved multiplex real-time PCR system developed by Roche Diagnostics, but even in this identification system, the sensitivity and specificity are not so accurate [58]. A comparison of diagnostic performance has been conducted among blood culture samples and SeptiFast, out of which 87.8% of culture-positive cases were detected by both blood culture and PCR. In a systematic review, a meta-analysis was performed, and a specificity of 0.86 and a sensitivity of 0.68 were observed [59]. Magicplex^TM^ is another type of multiplex real-time PCR developed by Seegene that can detect more than 90 microorganisms at their gene level, but its low sensitivity (29%) and specificity (95%) can limit its use in clinical applications [60]. Future challenges of PCR include the need for more standardized protocols, improvements in sensitivity and specificity, and addressing the limitations posed by complex and diverse microbial communities.

### 2.3. Mass Spectrometry

**Figure 3:**
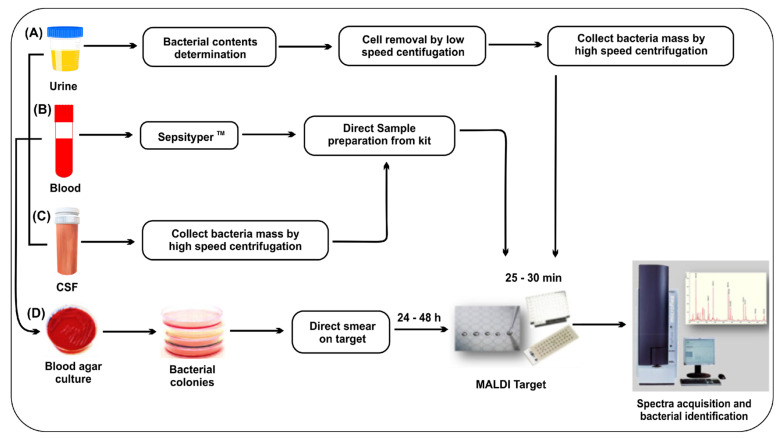
Workflow of MALDI-TOF-MS for bacterial identification. Some liquid samples such as (**A**) urine, (**B**) positive blood culture bottles, and (**C**) cerebrospinal fluid can be applied for direct identification following some sample preparation and extraction protocol, and (**D**) bacterial culture from standard agar plate. Reproduced with permission from [67]. Copyright 2020, Elsevier.

Mass spectrometry (MS) rapidly identifies bacteria in patient blood by detecting the mass-to-charge ratio (*m*/*z*) of ionized biological molecules, such as bacterial proteins [61,62]. The matrix-assisted laser desorption time of flight mass spectrometry (MALDI-TOF-MS) method is approved by the US FDA for identifying bacteria [63]. MALDI-TOF-MS basically detects bacteria on the basis of housekeeping genes and ribosomal proteins, which revolutionized bacteria identification in clinical laboratories [64,65]. It is possible to identify bacteria accurately and quickly, separating methicillin-resistant staph (MRSA) *Staphylococcus aureus* from methicillin-sensitive *Staphylococcus aureus* [66]. MALDI-TOF-MS can identify pathogens in less than an hour from purified bacterial pellets and commercialized kits such as the MALDI Biotyper Sepsityper^TM^ Kit (Figure 3) [67]. The bacterial sample can be obtained from various sources, such as clinical samples (urine, blood, cerebrospinal fluid) or culture plates. Sepsityper^TM^ is an easy-to-use sample preparation kit for the rapid identification of bacteria from positive blood cultures. It is designed to improve accuracy and simplify the process of sample preparation. The sample is mixed with a matrix to create a dried spot on a MALDI plate, which allows for the ionization of the bacterial sample during mass spectrometry [68,69].

A number of studies are also going on to combat the problem of antimicrobial resistance using MALDI-TOF-MS. The presence of β-lactam resistance can be conferred by MALDI-TOF-MS as it gives key spectral peaks corresponding to enzymatic modifications conferring antimicrobial resistance [70]. The identification of antibiotic resistance using MALDI-TOF-MS was observed through β-lactam ring hydrolysis after exposing the antibiotics to β-lactamase-producing bacteria, which revealed a decrease in the mass spectral peak of the antibiotic and the appearance of new peaks corresponding to its hydrolysis products [71]. There are some limitations to the technique. The coverage of the database is limited, as it may not contain all strains and species of bacteria. This can lead to misidentification [72,73]. A second issue could be interference during sample preparation. This can lead to inaccurate identification of contaminants or microorganisms in the sample [74]. MALDI-TOF-MS can also have trouble distinguishing closely related species or strains with similar mass spectra. To address this, it is possible to expand reference databases by including more strains and species of bacteria, develop new sample preparation techniques to reduce contamination, and use complementary methods, such as DNA sequences or phenotypic analysis, to confirm or resolve inconclusive results. MALDI-TOF-MS can be improved by developing new matrix formulations, improving instrumentation and data analysis algorithms, and integrating with other technologies.

### 2.4. Nanomaterials Based Detection

The use of nanomaterials for bacteria detection has attracted more attention in recent years. Nanoparticles can be used to detect particular bacteria based on their changed properties [75]. Nanoparticle-based detection methods could offer a number of advantages over traditional methods for bacteria detection in terms of sensitivity, specificity, and time for detection [76]. SPR is a unique optical property of noble metal nanoparticles that is a consequence of resonance due to the interaction of the collective oscillation of conduction band electrons of metal nanoparticles with incident light [77]. SPR of metallic nanoparticles can be fine-tuned over a broad spectral range (ultraviolet (UV) to near infrared (NIR)) and depends strongly on the particle shape, size, composition, and surrounding medium [78]. Plasmon-enhanced spectroscopy, such as surface-enhanced Raman scattering (SERS), is a result of the amplification of the local electromagnetic field due to SPR excitation [79]. Plasmonic metal nanoparticles have become important in biosensing as a result of advancements in nanofabrication techniques. Various platforms for bacteria detection could be developed using metallic nanoparticles, such as colorimetric and fluorescent detection platforms [80]. Among the metallic nanoparticles, gold nanoparticles (AuNPs) are the most widely utilized for colorimetric detection due to various advantages such as controlled synthesis, excellent solubility, and easy surface modification [81]. Target-induced colorimetric change is generally visible to the naked eye for qualitative detection or quantifiable by UV–visible spectroscopy. Priyadarshi et al. have demonstrated the impact of the size of AuNPs in colorimetric bacterial sensing where smaller AuNPs (20 nm) showed more sensitivity as compared to large sized AuNPs (40 nm) [33]. In another paper, Miranda et al. have reported a colorimetric assay using an enzyme-nanoparticle conjugate system for *E. coli* detection [82]. In this work, AuNPs were functionalized with quaternary amines electrostatically bound to β-galactosidase, inhibiting its activity. The enzyme activity was restored after its release from the nanocomplex, following AuNPs binding with *E. coli*, leading to an enhanced colorimetric readout. In this assay, 10^2^ bacteria/mL was the limit of detection in solution and 10^4^ bacteria/mL on a test strip. Similarly, Peng et al. demonstrated the detection strategy for various bacterial species on the basis of interactions between bacteria and phages [83]. The phage’s attachment to the bacterial surface and subsequent AuNP aggregation on the capsid resulted in a clear colorimetric change with a detection limit of 100 cells. Li et al. developed a colorimetric sensor array for the identification of 12 bacteria and 3 fungi, where four different types of functionalized AuNPs have been used as sensing elements [84]. The rapid color change was observed within 5 s due to the interaction between AuNPs and bacteria, which gave a unique color shift pattern. Fluorescence-based methods are more sensitive (up to 1000 times) as compared to colorimetric methods [85]. The amount of emitted light is directly proportional to the target analyte concentration in the sample, which can detect even low concentrations of analyte. Yin et al. used upconversion fluorescent nanoparticles for simultaneous detection of seven bacteria [86]. The construct was based on guanidium-functionalized upconversion fluorescent nanoparticles, hydrogen peroxide, and tannic acid, which quantify bacteria in a non-specific manner as bacterial presence effectively strengthens the nanoparticles’ luminescence. The proposed strategy was time saving, highly sensitive, and cost effective compared to the traditional approach. In another report, Phillips et al. developed a fluorescence-based biosensor for bacteria sensing using polymer-conjugated AuNP constructs, where polymers and AuNPs are used as flares and quenchers, respectively [87]. The anionic polymer conjugated with cationic AuNPs acted as fluorescence-quenched complexes. Upon bacteria addition, the polymer is released due to interaction between the anionic bacterial surface and cationic AuNPs, which results in fluorescence recovery. Another study by Zheng et al. utilized a silica-quantum dot-based fluorescent lateral flow immunoassay for simultaneous detection of *E. coli* and *S. typhimurium* [88]. The nanotags were directly mixed with the sample and loaded on the test strip, which showed a detection limit of 50 cells/mL within 15 min. Similarly, Yu et al. used vancomycin-functionalized gold nanoclusters and aptamer-functionalized AuNPs as energy donors and acceptors, respectively, for *S. aureus* detection [89]. This strategy showed a detection limit of 10 CFU/mL. Apart from bacterial detection at early stages, bacterial clearance is also important in sepsis patients. Various magnetic nanoparticle-based approaches have been developed recently for this purpose [90,91]. Lee et al. have developed synthetic ligand (zinc-coordinated bis(dipicolylamine))-modified magnetic nanoparticles for the removal of bacteria and their endotoxins from whole blood using a microfluidic device [92]. The ligand forms complexes with specific lipids present on the bacterial outer membrane, providing high binding affinity to pathogenic bacteria in the blood. The bacteria bound to modified magnetic nanoparticles were removed using magnetic microfluidic devices, resulting in 100% bacteria removal from bovine whole blood. Similarly, Shi et al. reported hemocompatible magnetic nanoparticles that bind and remove bacteria and their endotoxins circulating in blood without significantly affecting blood cells [93]. The nanoparticles were made hemocompatible using polydopamine coating and modified with an imidazolium-based ionic liquid, which possesses anti-bacterial activity. These studies provide a new platform for pathogen removal from blood that can be further improved for clinical use. The development of various nanosystems for bacteria detection is currently an active and growing area of research. Despite a lot of progress in recent years, there are still some challenges, such as their stability and toxicity, that need to be addressed before using nanoparticles in real-time clinical settings. The non-specific aggregation in complex biological systems can give false negative or positive results, which could be further improved by making the nanosystems more specific towards their target using different biorecognition molecules.

### 2.5. Surface Enhanced Raman Spectroscopy (SERS)

**Figure 4:**
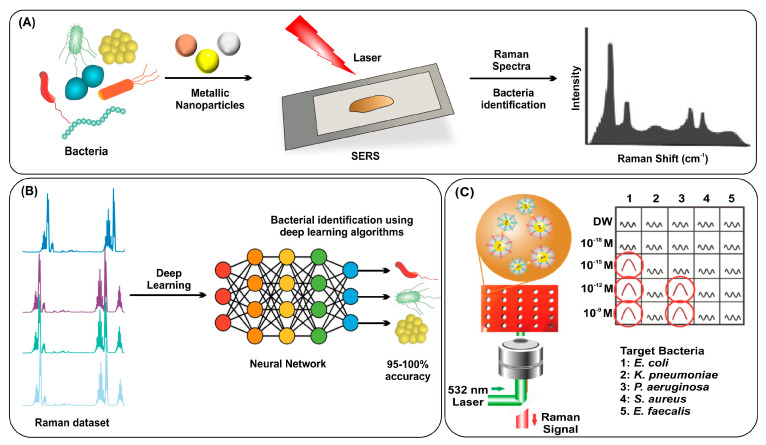
(**A**) Workflow for bacterial identification using SERS, (**B**) workflow for accurate bacterial identification using deep-learning strategy, and (**C**) measurement of Raman signals from the solutions in a multi-well array showing specificity for target bacteria. Reproduced with permission from [97,104,110]. Copyright 2022, American Chemical Society; copyright 2020, Wiley; copyright 2021, Elsevier.

Raman scattering is vibrational spectroscopy based on the interaction of light with matter, resulting in a unique spectral fingerprint that can identify the bacterial species [94]. Raman signal intensity could be greatly amplified by the phenomenon called SERS. For SERS, it is essentially required to use plasmonic nanomaterials with diverse geometries (Figure 4A) [95,96,97]. SERS could offer several benefits for bacteria identification, such as high sensitivity, specificity, multiplexing capability, and rapid analysis [98]. However, the spectral patterns are so complex that they cannot easily distinguish the bacteria species; therefore, algorithm-based analysis has been incorporated for accurate bacterial identification [99,100]. Deep-learning algorithms can accurately process complex patterns of spectra data, which can distinguish bacterial strains with high reliability [101,102]. In a recent study, a Raman spectra dataset with deep learning accurately identified 30 bacterial pathogens, differentiated antibiotic-susceptible bacteria with 89% accuracy, and achieved 99.7% accuracy for treatment identification [103]. Another study achieved 95–100% accuracy in identifying 12 different microbes with a deep-learning platform using the Raman dataset (Figure 4B) [104]. Ding et al. used SERS spectra of three Salmonella serovars to train a convolutional neural network, which enabled accurate identification of the three serovars with individual accuracy rates of 98.68%, 95.35%, and 96.17%, respectively [105]. Bacterial cellulose nanocrystals conjugated with Concanavalin A lectin were used for *E. coli* identification. A set of supervised learning models called support vector machines (SVM) was used for classification in this study and achieved 87.7% accuracy in discriminating 19 bacterial strains [106]. Another study showed that SERS and machine learning can rapidly identify bacterial susceptibility to antibiotics with over 99% accuracy in 10 min. Bayesian Gaussian mixture analysis offers a promising approach toward practical, rapid antimicrobial susceptibility testing [107]. The technique demonstrated strong 97.8% accuracy, with the k-nearest neighbors algorithm exhibiting superior performance [108]. A new deep-learning model called the dual-branch wide kernel network has been used to boost the efficacy of SERS for detecting *E. coli* and *S. epidermidis* in media without the need for separation procedures. This technique has shown classification accuracies of up to 98%, making it a fast and effective method [109].

Label-based SERS methods use tags with Raman reporter molecules that bind to target bacteria, providing sensitive and unique signals. Particle labeling significantly improves bacterial identification using SERS. A study demonstrated that using solution state gold silver core–shell nanodumbbells with target nucleic acid significantly improved the signal reproducibility of SERS for bacterial identification, achieving a higher sensitivity of 4.5 CFU/mL compared to culture-based assays and conventional PCR (Figure 4C) [110]. SERS can help identify bacteria in complex samples with low pathogen concentrations. Magnetically assisted SERS based on aptamer recognition identified Staphylococcus aureus at 10 cells/mL [111]. Similarly, gold nanoflowers were used to design a self-calibrating SERS system for identifying bacterial phenotypes via specific DNA sequences, achieving a detection limit of around 5 fM for *S. aureus* DNA identification [112]. The incorporation of magnetic microparticles with SERS showed excellent sensitivity for bacterial identification compared to fluorescence by minimizing the non-specific binding of NPs during the target binding step [113]. Combining magnetic material and SERS label for immunoassay was reported for bacterial identification with limits of 10–25 cells/mL for *E. coli*, *L. monocytogenes,* and *S. typhimurium* [114]. Simultaneous bacterial analysis and eradication via photothermal treatment were also reported by Gao et al. They demonstrated the SERS platform for offering potential anti-bacterial applications [115]. SERS spectra limitations have led to the development of electrochemical surface-enhanced Raman spectroscopy, allowing for more efficient differentiation of bacterial species in a biologically relevant electric field environment [116].

These recent results collectively demonstrate that SERS-based bacterial identification is a promising platform for accurately identifying target bacteria. With the continued advancements in nanotechnology and machine-learning algorithms, SERS is expected to be a reliable and rapid tool for the identification of bacteria, particularly in clinical settings where rapid and accurate diagnosis is crucial for patient outcomes.

## 3. Methods to Destroy Bacteria

### 3.1. Antibiotics

Antibiotics are drugs that are used to treat bacterial infections and are critical for managing and treating a number of bacterial infections [117,118,119,120]. In the past few decades, antibiotics have been widely used in anti-bacterial applications; however, the excessive use of antibiotics results in the development of multidrug resistance in bacteria. The emergence of antibiotic-resistant bacteria has become an alarming concern worldwide [121]. Antibiotics should be used judiciously and according to evidence-based guidelines to minimize the risk of resistance. It is important to weigh the benefits, potential side effects, and adverse reactions associated with antibiotics, such as allergic reactions and gastrointestinal disturbances. In addition, the individual patient’s medical history, comorbidities, and risk factors must be considered when prescribing antibiotics. Due to their side effects, antibiotics should be prescribed only when they are necessary and effective and for the shortest possible duration.

Antibiotics are chemical compounds that are generally produced by one kind of bacteria and, in higher quantities, can be used to kill other bacteria. The first synthesized antibiotic was arsphenamine (also known as salvarsan), which was launched in the 1910s to cure syphilis. It was widely used to treat syphilis infections until penicillin was discovered as a natural antibiotic [122]. The golden age of natural antibiotics started after the discovery of penicillin. Currently, there are various kinds of antibiotic substances available for different bacterial infections [123,124]. Based on their chemical structures, there are eight classes of antibiotics that include beta-lactams [125], macrolides [126,127], tetracyclines [128,129], quinolones [130], aminoglycosides [131], sulfonamides [132], glycopeptides [133,134], and oxazolidinones [135,136].

Although effective antibiotics are available for bacterial infection treatment, due to the uncontrolled use of antibiotics and their use for agricultural purposes, bacteria are gaining resistance to these available antibiotics. This antibiotic resistance leads to the development of antibiotic-resistant bacteria such as *Staphylococcus aureus*, drug-resistant *Salmonella typhimurium*, and multidrug-resistant *Pseudomonas aeruginosa*. It has been observed that bacteria acquire antibiotic resistance by programming their cellular pathways through alternative pathways. The resistant bacteria tackle the beta-lactam antibiotics by mechanisms such as hydrolyzation of the antibiotics by beta-lactamases, alteration in the molecular structure of penicillin-binding proteins, synthesis of efflux pump proteins for ejection of antibiotics from the bacterial cells, shielding of ribosomes by ribosome protection proteins, tampering with the antibiotic’s structure, and mutations in the dihydropteroate genes [137].

### 3.2. Nano-Biotechnologies

Recently, nanobiotechnology approaches have been widely explored for the treatment and eradication of bacterial infections. A number of nanoparticles have been well studied by different research groups for their anti-bacterial activity. The anti-bacterial mechanism of nanoparticles can be due to metal ion release at the targeted site, oxidative stress, or a non-oxidative mechanism. A combination of all mechanisms can also be collectively responsible for killing bacteria. These three types of mechanisms can occur simultaneously. NPs such as polymers, metals, and carbon NPs have been shown to possess anti-bacterial properties.

#### 3.2.1. Polymeric Nanostructures

**Figure 5:**
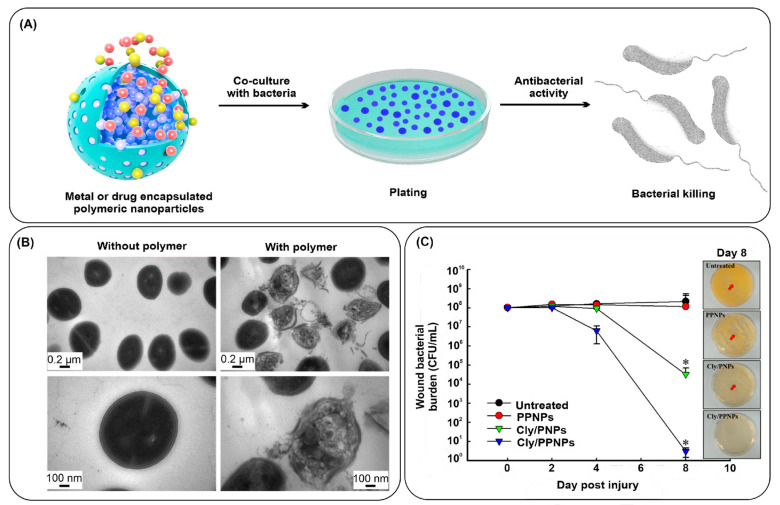
(**A**) Schematic representation of anti-bacterial activity of metal or drug encapsulated polymeric NPs; (**B**) Comparative TEM images of *E. faecalis* in the absence and presence of synthesized polymer; and (**C**) the number of bacteria present on wounds; swab culture was used for checking bacteria load at day 8 after injury (* indicate *p* < 0.05 compared with untreated group). Reproduced with permission from [139,142,143]. Copyright 2018, Frontiers; copyright 2014, Royal Society of Chemistry; copyright 2019, MDPI.

Several studies have evaluated the application of polymers in anti-bacterial therapies. Polymers do not possess anti-bacterial properties; however, they are widely used as vector molecules to deliver anti-bacterial drugs to their target sites. Chitosan is one of the polymers with anti-bacterial properties. Properties such as cytocompatibility, enhanced drug adsorption, solubility, and controlled drug release make the polymeric nanostructures suitable for bactericidal applications. Polymers can be structured into nanoparticles and small and large-sized vesicles [138]. Various precursors can be utilized to form polymers, such as natural polymers (chitosan) or synthetic polymers (polylactic-co-glycolic acid (PLGA) and polylactic acid (PLA)). Diverse polymers can be used for the encapsulation of metal and drug nanoparticles possessing anti-bacterial activities (Figure 5A) [139].

Chitosan has an intrinsic anti-bacterial property due to its positively charged surface. There is electrostatic interaction between positively charged chitosan molecules and negatively charged bacterial membranes. This facilitates the change in membrane permeability, ultimately leading to bacterial killing. Various studies have utilized this property of chitosan in formulating chitosan-based nanostructures for anti-bacterial applications. In a recent study by Ejaz et al., mannose-modified chitosan was synthesized. The developed nanostructures were able to efficiently kill both Gram-positive and Gram-negative bacteria [140]. Furthermore, Kritchenkov and co-workers have demonstrated the chemical modifications in the chitosan structure to develop a betaine derivative of chitosan through ultrasound-assisted catalyst-free thiol-yne click chemistry. The constructed derivative’s anti-bacterial affinity was checked against the commercially available drugs ampicillin and gentamicin. Surprisingly, the nanoparticles showed higher anti-bacterial characteristics than the commonly used drugs [141]. A cationic polymer micelle has been synthesized for its anti-bacterial effect against drug-resistant Gram-positive bacteria [142]. After the treatment of bacteria with the polymeric micelles, clear damage in the cell wall as well as in the membrane of *E. faecalis* is clearly visible (Figure 5B). Similarly, in another report, clindamycin-encapsulated polymeric nanoparticles have been prepared for anti-bacterial effect against MRSA [143]. The synthesized drug-loaded nanoparticles, when applied to wounds, have shown a great reduction in the bacterial burden at day 8 post-injury (Figure 5C).

Apart from natural chitosan polymers, synthetic polymers such as PLA and PLGA have been extensively explored for efficient drug delivery. Many studies have demonstrated its use as a core material to functionalize the ligand molecule to target bacteria. For example, Ucak and co-workers have tried to overcome antibiotic resistance by targeting specific bacteria, reducing the high dosage demand, and ultimately speeding down antibiotic resistance evolution. In this study, they utilized the specific aptamers against *S. aureus* for targeted delivery of teicoplanin antibiotics from PLGA nanoparticles [144]. Furthermore, other studies have also evaluated the anti-bacterial performance of PLGA-based nanoparticles and their efficiency in the delivery of antibiotics [145] and other active bactericidal biomolecules [146]. Da Costa et al. have probed the antibiofilm character of PLA nanoparticles and their efficiency in delivering rifampicin antibiotics. They have modified the outer surface of PLA nanoparticles with poly-L-lysine to disrupt the bacterial membrane by utilizing the positive charge of poly-L-lysine [147]. In another study, PLA nanoparticles were synthesized to encapsulate an essential oil to be used against both Gram-negative and Gram-positive bacteria [148].

#### 3.2.2. Noble Metal Nanoparticles

**Figure 6:**
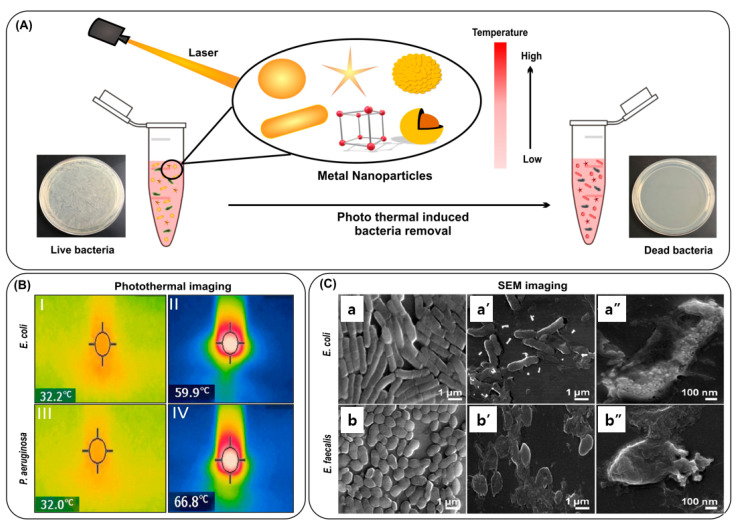
(**A**) Schematic representation of photothermal ablation of bacteria incubated with metallic nanoparticles in the presence of NIR; (**B**) photothermal image of *E. coli* and *P. aeruginosa* with mannose functionalized AuNRs, showing temperature before (I, III) and after treatment (II, IV); and (**C**) scanning electron microscopy (SEM) images of *E. coli* and *E. faecalis* where (**a**,**b**) are SEM images of untreated cells, and (**a′**,**b′**,**a″**,**b″**) are dead bacteria, post NIR treatment. Reproduced with permission from [160,162]. Copyright 2019, Elsevier; copyright 2014, Royal Society of Chemistry.

Noble metal nanoparticles have a unique SPR property, which is the origin of strong photothermal effects and other optical phenomena. This strong photothermal effect can be utilized for bacteria removal with light illumination, as shown in Figure 6A [149,150,151]. The photothermal effect can destroy bacteria with strong thermal damage. In addition to the photothermal effect, the photodynamic effect can further improve the death of bacteria by generating highly reactive oxygen species, which require the use of photosensitizers [152,153,154].

When irradiated for five minutes with an 808 nm laser, polygonal AuNPs conjugated to vancomycin killed more than 99% of bacteria [155]. Researchers have developed AuNPs with pH-responsive self-adaptive properties, resulting in enhanced photothermal injury and minimal damage to healthy cells [156]. Gold nanorods (AuNRs) have been used for PTT because of their high absorption property in the NIR wavelength, which leads to an efficient conversion of photons into heat [157]. Proper surface modifications are also helpful to further improve the performance [158,159]. AuNRs combined with carbohydrates were used to kill bacteria efficiently [160]. Figure 6B shows that during photothermal ablation with AuNRs, the temperature rose from 32.2 °C to 59.9 °C for *E. coli* and from 32.0 °C to 66.8 °C for *P. aeruginosa*. The photothermal activity could be increased by coating AuNPs with r-GO (reduced graphene oxide), leading to more efficient cell death [161]. The use of AuNRs coupled with magnetic nanoparticles for bacterial ablation has also been reported [162]. The AuNRs could kill bacteria with thermal energy, facilitating a separation via magnetic nanoparticles for their reusability in subsequent cycles (Figure 6C).

Inorganic materials, such as silver nanoparticles, copper sulfide, and iron oxide nanoparticles, can act as anti-bacterial and photothermal agents, with the potential to disrupt bacterial membranes and release anti-bacterial ions [163,164,165]. Silver nanoparticles (AgNPs) embedded on polyethyleneimine-functionalized glass surfaces have been shown to have enhanced anti-bacterial effects on *S. aureus* and *E. coli* through both internal and near-infrared photothermal mechanisms [166]. CuS nanoparticles have been studied as an anti-bacterial agent but have only recently been explored for their potential as a photothermal agent. A multimodal nanomaterial synthesized by embedding CuS nanoparticles onto NaYF (Mn/Yb/Er@photosensitizer) with SiO_2_ was demonstrated to effectively remove *E. coli* and *S. aureus* through photodynamic and photothermal mechanisms [167]. Additionally, iron oxide NPs have been reported to effectively eliminate Gram-positive and Gram-negative bacteria through photothermal treatment with recyclable FeO [168].

Due to their large surface and multiple functional moieties, 2D nanoparticles also hold great promise in the field of sensing and diagnosis [169]. Molybdenum disulfide (MoS_2_) nanoparticles have potential as a biocompatible photothermal agent due to their ability to absorb a broad range of NIR [170]. PEG-MoS_2_ nanoflowers have shown potential anti-bacterial activity against ampicillin-resistant *E. coli* and *B. subtilis* [171]. Silver nanocubes on nanosheets of molybdenum dioxide have been engineered to act as photothermal agents for the treatment of bacteria [172]. Graphene and its derivatives exhibit strong plasmonic resonance in the mid- and far-infrared regions, making them useful for photothermal therapy [173]. Reduced graphene oxide has shown six times more NIR absorbance than non-reduced graphene oxide, allowing for effective photothermal ablation of cells even at low concentrations [174]. rGO-PEG-AuNRs have been shown to enhance photothermal activity, leading to 99% bacterial killing in 10 min when used against uropathogenic *E. coli* UTI89 [175].

#### 3.2.3. Bacterial Killing by Mechanical Rupture

**Figure 7:**
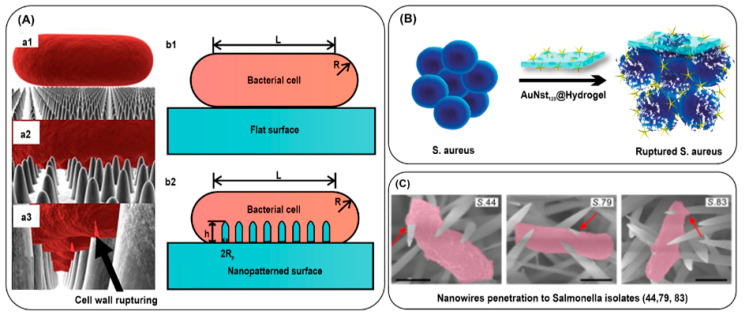
(**A**) Three-dimensional representation of the modeled interactions between a rod-shaped cell and the wing surface. The cell into contact (**a1**), adsorb onto the nanopillars (**a2**), and rupturing of cell wall by nanopillars (**a3**). Bacterial cell on the flat surface (**b1**) or nanopatterned surface (**b2**). (**B**) Schematic representation of rupturing of *S. aureus* after incubation with gold nanostars embedded in hydrogel, and (**C**) representative SEM images of the BLS *Salmonella* (isolates 44, 79, and 83) appeared to be significantly penetrated through interaction with the sharp NWs. Reproduced with permission from [176,181,182]. Copyright 2017, Elsevier; copyright 2022, American Chemical Society; copyright 2020, American Association for the Advancement of Science. Scale bars, 500 nm.

In other ways, metal nanostructures are so rigid that they can damage the cell membrane or cell wall of bacteria, which results in the complete killing of bacteria. This approach is also effective depending on numerous aspects such as the size, shape, and concentration of nanoparticles and the frequency and intensity of the mechanical stimulus. A class of nanomaterials with sharp spikes possesses unexplored, multifaceted characteristics. Many natural nanostructured surfaces possess a bactericidal effect when they come into contact with bacterial surfaces. Such naturally occurring as well as bio-inspired nanostructured surfaces have been reviewed in a study [176]. Many physical models have been reported for the bacterial interaction with nanostructured surfaces (Figure 7A). Additionally, some research groups have also investigated the use of insects to construct natural surfaces that can limit bacterial contamination [177]. A study showed that the wings of *Psaltoda claripennis* cicada can be used as an antibiofouling surface due to their hydrophobicity and self-cleaning characteristics [178]. Shimada and coworkers prepared nanowires of zinc oxide coated with silicon dioxide (ZnO/SiO_2_) and evaluated their mechanical effect on *E. coli*. The study showed that the nanowires were able to inactivate almost 99% of *E. coli* [179]. Moreover, it has been observed that gold nanostructures such as star-shaped nanoparticles can have an anti-bacterial effect due to the membrane-puncturing ability of the synthesized nanospikes [180]. Kaul et al. unraveled the mechano-bactericidal action of star-shaped gold nanoparticles and hydrogel (Figure 7B) [181]. The gold nanostars of 120 nm spike length were efficient for killing bacteria. In this case, more than 95% killing was observed in *P. aeruginosa* and *E. coli*, with a further 60% killing in *S. aureus*. When using star-shaped gold nanoparticles with hydrogel, it showed a reduction of >90% of colonies of *P. aeruginosa* and *E. coli*. In comparison, around 35.4% of colonies are obtained for *S. aureus*. Similarly, in one more study, the mechanical penetration of nanowires has been compared among β-lactam resistant and susceptible bacteria, and the effective penetration efficiency of nanowires has been demonstrated in β-lactam susceptible bacteria (Figure 7C) [182].

Although diverse strategies for using nanoparticles have shown great progress in bacterial killing, some challenges, such as toxicity, need to be addressed for their safe use in clinical settings. Some of the examples of nanomaterials for bacterial killing have been summarized in Table 2 on the basis of action mechanisms.

**Table 2:** Examples of nanomaterials as anti-bacterial agents based on the action mechanisms.

Mechanism of Action	Nanomaterial	Target	Scope	Limitations	Ref.
Antibiofilm activity	Mannose-functionalized chitosan nanosystems	*E. coli* *L. monocytogenes* *P. aeruginosa* *S. aureus*	Effective against both Gram-positive and Gram-negative bacteria.	The biocompatibility of the nanosystems has not been clearly explained.	[140]
Sustained release of antibiotics	Clindamycin-loaded polymeric particles	MRSA	Antibiotic-loaded NPs accelerate MRSA-infected wounds.	Only applicable for the treatment of topical infections.	[143]
	Teicoplanin-encapsulated aptamer-functionalized PLGA NPs	*S. aureus* strains	Aptamers provide specificity against *S. aureus*, while PLGA NPs decrease the MIC value for teicoplanin.	The biocompatibility of the functionalized nanoparticles has not been clearly explained.	[144]
PTT/PDT	Thiol chitosan-wrapped gold nanoshells	*E. coli* *P. aeruginosa* *S. aureus*	PTT effectively eradicates both Gram-negative and Gram-positive bacteria within 5 min.	Only applicable for the treatment of topical infections.	[150]
	Thiol-coated gold Nanostars	*E. coli* *S. aureus*	Hyperthermia results from PTT kill 99.99% of bacteria.	The biocompatibility of the functionalized nanostars has not been clearly explained and only applicable for the eradication of bacteria from medical devices.	[151]
	Galactose-modified porphyrin-conjugated gold NPs	*P. aeruginosa*	Galactose gives the specificity against *P. aeruginosa* and porphyrin eliminates bacteria via PDT and PTT.	The nanomaterial was able to eradicate only 70% of bacteria (colony count method) and only applicable for the treatment of topical infections.	[154]
	pH-responsive gold nanoparticles	MRSA biofilm	AuNP aggregation within the biofilm enhanced the photothermal ablation of MRSA.	Only applicable for the treatment of topical infections such as wound in MRSA infection.	[156]
	Glycoconjugate-coated gold nanorods	*E. coli* *P. aeruginosa*	Hyperthermia results from PTT against Gram-negative bacteria.	The higher temperature rise due to the gold nanorods may affect the normal tissues if used for the treatment of bacterial infections.	[160]
	Gold nanorod-conjugated magnetic nanoparticles	*E. coli* *E. faecalis*	Recyclable nanocomposite for repeated photothermal effect.	No in vivo studies were performed to show the safety and efficacy of nanocomposite.	[162]
	Silver nanoplates	*E. coli* *S. aureus*	Anti-bacterial properties and PTT effect synergistically eradicate bacteria.	Cytotoxicity and biocompatibility must be calculated for its real time application in clinical settings.	[166]
	UCNP/PS (upconversion nanoparticles with photosensitizers)	*E. coli* *S. aureus*	PTT and PDT effectively eradicate both Gram-negative and Gram-positive bacteria.	The biocompatibility and cytotoxicity of the functionalized nanoparticles have not been clearly explained and applicable for only topical application.	[167]
Mechanical rupture	ZnO/SiO_2_ nanowires	*E. coli*	ZnO/SiO_2_ nanowires inactivate 99% of *E. coli* inactivation.	Only effective for Gram-negative bacteria.	[179]
	Gold nanostar-based hydrogel	*E. coli* *P. aeruginosa* *S. aureus*	Nanospikes of gold nanostars rupture the bacterial membrane.	Limited anti-bacterial effect in the Gram-positive bacteria *S. aureus.*	[181]
	NiCo(OH)_2_CO_3_ nanowires	*Salmonella* *E. coli* *P. aeruginosa* *K. pneumoniae*	Nanowires mechanically penetrate the bacterial cell envelope.	Only effective for Gram-negative bacteria.	[182]

## 4. Conclusions and Future Directions

Rapidly identifying pathogenic bacteria before sepsis occurs is crucial for saving a patient’s life and further preventing its occurrence. Bacteria have adapted their intrinsic abilities to combat antibiotic treatments, raising the problem of antimicrobial resistance. In this review, nanotechnology-based approaches have been covered for bacterial identification and killing. Among the diverse areas of application of nanotechnology, it has gained growing attention in bacteria identification and anti-bacterial applications based on the unique optical and physicochemical properties of nanomaterials. Bacterial identification through conventional culture-based methods suffered from limitations such as being time-consuming and requiring trained personnel and instruments, which made the method less efficient for timely pathogen identification. The recently developed PCR and mass spectrometric methods offered improved performance in terms of assay time, but the accuracy for bacteria identification was not completely successful. In contrast, the SERS-based method offered many advantages in the identification of bacteria, which requires the use of plasmonic nanomaterials for signal enhancement. The accuracy of bacteria identification could be further improved by incorporating the algorithm-based analytical method. Although it is not a stage of clinical usage, it is expected the incorporation of new technology in the clinic in near future.

The unique properties of nanomaterials can open new areas of application for an efficient anti-bacterial treatment. Nanoparticles can deliver the anti-bacterial drugs selectively to the target bacteria, which can minimize the overuse of anti-bacterial drugs. The efficient photothermal properties of different nanomaterials can actively kill bacteria and destroy bacterial biofilm. The improved photodynamic property can also efficiently kill bacteria. The PTT and PDT properties of nanoparticles can be combined with antibiotics for further potent bacterial eradication. Mechanical rupture is also proven to be an effective way for bacteria to kill on the surface, which can be one way of minimizing the spread of bacteria.

In conclusion, nanomaterials with distinct physicochemical properties have been extensively investigated for accurate identification and killing of bacteria. In this review, we highlighted the recent advances in nano-biotechnologies for bacterial identification and anti-bacterial properties. It is expected that the new technologies can open new avenues of methods to address the limitations of conventional methods in the near future.

## Figures and Tables

**Table 1:** The performances of diverse technologies for bacteria identification.

Technique	Cost	Time	Sensitivity	Specificity	Scope	Limitations	Ref.
Culture	Low	Long	Low	High	Broad range of bacteria can be identified (both Gram-negative and Gram-positive), antibiotic susceptibility can be tested, familiar to most clinical facilities.	Time consuming, susceptible to contamination, low sensitivity for some bacterial species.	[47,48,49,50,51,52]
PCR	High	Short	High	High	Fast, sensitive, specific, can identify multiple bacteria simultaneously, and can quantify even small number of bacteria in real time.	False positives or negatives due to contamination, unable to differentiate among closely related bacterial strains due to complex bacterial genome sequences.	[53,54,55,56,57,58,59,60]
Mass spectrometry	High	Short	High	High	Sensitive, specific, and can identify broad range of bacteria directly from clinical samples, and can identify bacteria in low concentration.	Limited database coverage, expensive, requires specialized equipment and trained personnel.	[61,62,63,64,65,66,67,68,69,70,71,72,73,74]
Nanomaterial	Low	Short	High	High	Rapid, sensitive, specific, and can detect and quantify bacteria in real-time settings.	Sensitive to environmental factors such as temperature, pH, salinity, and non-specific aggregation in complex media.	[75,76,77,78,79,80,81,82,83,84,85,86,87,88,89,90,91,92,93]
SERS	Moderate	Short	High	High	Rapid, high sensitivity, and specificity due to unique spectral fingerprint, can identify bacteria in low concentrations, can identify broad range of bacteria in real time directly from clinical samples.	Requires specialized equipment, difficult to interpret spectra without deep-learning algorithms.	[94,95,96,97,98,99,100,101,102,103,104,105,106,107,108,109,110,111,112,113,114,115,116]
